# Joint Acidosis and GPR68 Signaling in Osteoarthritis: Implications for Cartilage Gene Regulation

**DOI:** 10.3390/genes17010109

**Published:** 2026-01-20

**Authors:** Colette Hyde, Adam Yung, Ryan Taffe, Bhakti Patel, Nazir M. Khan

**Affiliations:** 1 Department of Orthopaedics, Emory Musculoskeletal Institute, Emory University, Atlanta, GA 30329, USA; colette.hyde@emory.edu (C.H.); adam.yung@emory.edu (A.Y.); ryan.taffe@emory.edu (R.T.); bpatel331@gatech.edu (B.P.); 2 College of Sciences, Georgia Institute of Technology, Atlanta, GA 30332, USA; 3 Joseph Maxwell Cleland Atlanta VA Medical Center, Decatur, GA 30033, USA

**Keywords:** GPR68, osteoarthritis, joint acidosis, proton-sensing GPCR, cartilage inflammation, extracellular pH, synovial inflammation

## Abstract

Joint acidosis is increasingly recognized as an important determinant of cellular behavior in osteoarthritis (OA). Declines in extracellular pH (pHe) occur across cartilage, meniscus, synovium, and subchondral bone, where they influence inflammation, matrix turnover, and pain. Among proton-sensing G protein-coupled receptors, GPR68 responds to the acidic pH range characteristic of human OA joints. The receptor is activated between pH 6.8 and 7.0, couples to Gq/PLC-MAPK, cAMP-CREB, G12/13-RhoA-ROCK signaling pathways, and is expressed most prominently in articular cartilage, with additional expression reported in synovium, bone, vasculature, and some neuronal populations. These pathways regulate transcriptional programs relevant to cartilage stress responses, inflammation, and matrix turnover. GPR68 expression is increased in human OA cartilage and aligns with regions of active matrix turnover. We previously reported that pharmacologic activation of GPR68 suppresses IL1β-induced MMP13 expression in human chondrocytes under acidic conditions, indicating that increased GPR68 expression may represent a microenvironment-responsive, potentially adaptive signaling response rather than a driver of cartilage degeneration. Evidence from intestinal, stromal, and vascular models demonstrates that GPR68 integrates pH changes with inflammatory and mechanical cues, providing mechanistic context, although these effects have not been directly established in most joint tissues. Small-molecule modulators, including the positive allosteric agonist Ogerin and the inhibitor Ogremorphin, illustrate the tractability of GPR68 as a drug target, although no GPR68-directed therapies have yet been evaluated in preclinical models of OA. Collectively, current data support GPR68 as a functionally relevant proton sensor within the acidic OA joint microenvironment.

## 1. Introduction

Osteoarthritis (OA) is a progressive joint disease characterized by pain, stiffness, and structural degeneration, affecting hundreds of millions of individuals worldwide [1,2,3,4,5]. Although OA has historically been viewed as a consequence of mechanical wear and tear, it is now recognized as a complex disorder involving dysregulated interactions between mechanical loading, cellular metabolism, inflammation, and tissue repair processes. Major risk factors include aging, obesity, prior joint injury, occupational or sports-related loading, and sex-specific biological differences, all of which contribute to heterogeneous disease trajectories and clinical outcomes [6,7,8]. Despite its high prevalence and societal burden, disease-modifying therapies for OA remain limited, underscoring the need to identify microenvironmental cues that shape joint cell behavior and may offer new therapeutic entry points.

Mechanical and biomechanical factors play a central role in shaping OA risk and progression. Repetitive occupational loading, high-impact sports participation, and prior joint injury alter cartilage structure, extracellular matrix organization, and tissue mechanics, predisposing joints to degeneration long before radiographic disease becomes evident [9,10,11]. Obesity further amplifies these effects through combined mechanical overload and metabolic stress, while sex-related differences in cartilage composition, joint alignment, and hormonal regulation contribute to distinct OA susceptibility and progression patterns [9,10,11]. Together, these factors highlight OA as a mechanically driven but biologically modulated disease and underscore the need for early, objective biomarkers capable of capturing joint dysfunction before irreversible structural damage occurs.

**Figure 1:**
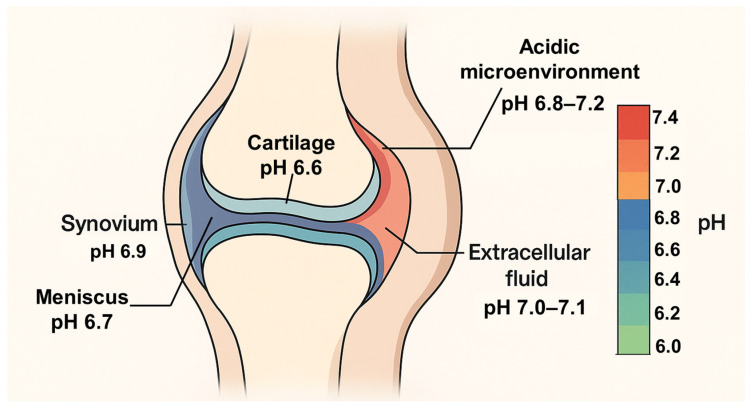
Schematic representation of extracellular pH gradients in the OA knee joint. Cross-sectional illustration showing cartilage and meniscus as more acidic (warmer colors) and synovial fluid as relatively less acidic (cooler colors), based on pHe ranges reported in acidoCEST-UTE MRI and synovial fluid studies. The color bar indicates approximate extracellular pH from 7.4 to 6.0, highlighting heterogeneous acidic microdomains that can engage proton-sensing receptors such as GPR68 in OA This schematic illustrates the spatial distribution of extracellular acidification within the OA joint. Reported pH ranges for cartilage, meniscus, and synovial fluid are based on acidoCEST-UTE MRI measurements in human knees, whereas other compartments are shown conceptually to illustrate potential acidic microenvironments.

Beyond biomechanics, increasing evidence indicates that the joint microenvironment itself plays an active role in OA pathophysiology. In particular, extracellular acidosis has emerged as a functionally relevant but underappreciated feature of the OA joint [12,13,14]. Recent advances in imaging have enabled direct visualization of joint acidosis in vivo. Using acidoCEST-UTE MRI, localized acidic microdomains have been identified in human OA cartilage and meniscus, with extracellular pH (pHe) values lower than surrounding synovial fluid and adjacent soft tissues [13,15]. Declines in pHe occur within cartilage, meniscus, synovial fluid, and subchondral bone, driven by altered metabolism, impaired nutrient diffusion, inflammation, and mechanical stress [12,13,14]. Figure 1 summarizes this heterogeneous pHe landscape across cartilage, meniscus, synovial fluid, and synovial lining, illustrating how localized acidic microdomains arise within the OA joint (Figure 1). Even modest reductions in pHe within the physiologic-to-pathologic range can suppress matrix synthesis, alter ion transport, and promote catabolic gene expression in chondrocytes, indicating that extracellular pH is not merely a by-product of inflammation but a biologically active signal that modulates cell function [16,17].

Acidic conditions also influence multiple joint-resident cell populations. Synovial fibroblasts and macrophages respond to low pH with enhanced cytokine and chemokine production, while sensory neurons express proton-sensitive ion channels that contribute to nociception and hyperalgesia in acidic environments [18,19]. Among the molecular systems capable of detecting extracellular acidosis are proton-sensing ion channels (such as ASIC3 and TRPV1) and a small family of proton-sensing G protein-coupled receptors (GPCRs), including GPR4, GPR65, GPR68, and GPR132 [14,20,21]. Whereas ASIC3 and TRPV1 have well-established roles in acid-induced joint pain [18,22,23], the functions of proton-sensing GPCRs in OA remain less clearly defined.

GPR68 (also known as OGR1) has gained particular attention because it is activated in the mildly acidic pH range relevant to OA cartilage and synovial fluid, and because it couples to signaling pathways that regulate inflammatory, catabolic, and stress responses [24,25]. Direct evidence supporting a role for GPR68 in OA is currently strongest in articular cartilage, where GPR68 expression is increased during disease progression and functional studies demonstrate that pharmacologic activation of GPR68 suppresses IL-1β-induced MMP13 expression in human chondrocytes under acidic conditions [26]. These findings suggest that GPR68 upregulation in OA cartilage may represent a microenvironment-responsive, potentially adaptive signaling mechanism rather than a primary driver of tissue degeneration. By contrast, evidence for GPR68 function in other joint compartments, including synovium, endothelium, and sensory neurons, is limited and largely inferred from studies in non-joint tissues such as intestine, lung, vasculature, and cancer models. Accordingly, interpretations of GPR68 activity outside cartilage should be considered hypothesis-generating rather than established mechanisms in OA.

The purpose of this review is to synthesize current knowledge on GPR68 as a proton-sensing receptor in the context of joint acidosis, with a primary focus on cartilage-relevant evidence. We summarize its pH-dependent activation properties and downstream signaling, evaluate compartment-specific evidence across joint tissues while explicitly distinguishing direct OA data from extrapolation, and discuss therapeutic implications and research priorities. By framing GPR68 as a context-dependent interpreter of extracellular acidosis rather than a universal driver of OA pathology, this review aims to provide a balanced foundation for future mechanistic and translational studies.

## 2. GPR68 as a Proton Sensor in the OA pH Range

**Figure 2:**
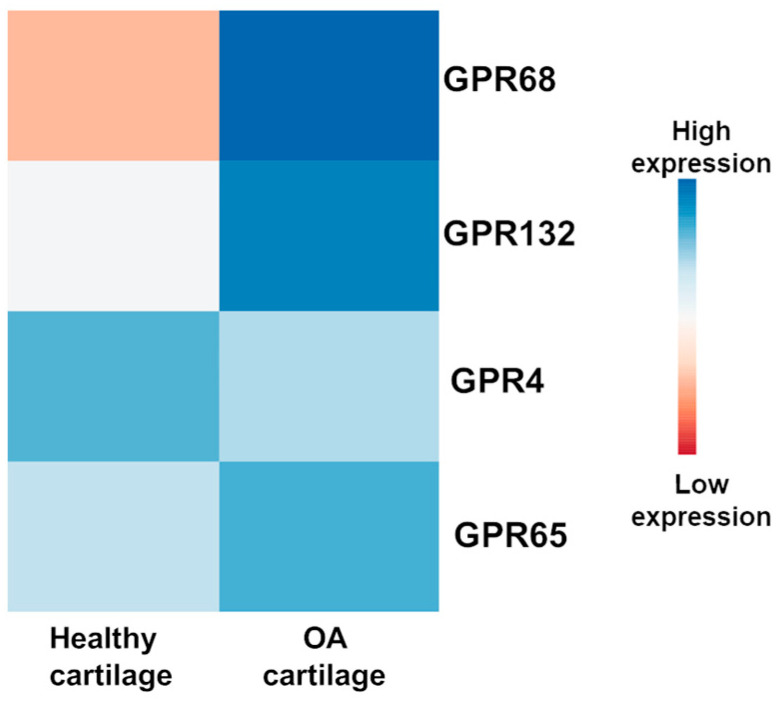
Qualitative ranking of proton-sensing GPCR expression in human cartilage. Heatmap summarizing relative expression of GPR4, GPR65, GPR68, and GPR132 in healthy and OA human knee cartilage, based on re-analysis of the public RNA-seq dataset GSE114007. Tiles indicate qualitative levels highlighting GPR68 as the most abundantly expressed and most strongly upregulated proton-sensing GPCR in OA cartilage. Colors represent normalized gene expression levels across samples, with higher expression indicated by blue colors. This analysis highlights relative transcriptional enrichment but does not establish functional activity or causal involvement in OA.

To determine which proton-sensing GPCRs are most relevant in cartilage, RNA-seq data from human knee cartilage (GSE114007) were re-analyzed, comparing OA cartilage to healthy controls. Among the four classical proton-sensing GPCRs (GPR4, GPR65, GPR68, GPR132), GPR68 showed the highest transcript abundance and the strongest upregulation in OA cartilage [27] (Figure 2). These expression data indicate transcriptional predominance but do not establish functional dominance or causal involvement in OA progression. These findings are consistent with our recent studies that increased GPR68 expression with OA severity and demonstrating that pharmacologic activation of GPR68 suppresses IL1β-induced MMP13 expression in human chondrocytes [26].

Multiple proton-sensitive receptors contribute to OA pathophysiology, particularly in the context of pain and inflammation. Acid-sensing ion channels such as ASIC3 and TRPV1 are well-established mediators of acid-evoked nociception and pain sensitization in OA and related musculoskeletal conditions [18,22,23]. In parallel, other proton-sensing GPCRs, including GPR4, GPR65, and GPR132, have been implicated in inflammatory and immune regulation in various tissues and disease contexts [14,20,21]. GPR68 is not presented here as the dominant proton sensor in OA; rather, it is discussed because of its enrichment in articular cartilage, activation within the pH range measured in OA joints, and ability to couple extracellular acidity to transcriptional programs relevant to cartilage homeostasis. These features distinguish GPR68 mechanistically from acid-sensing ion channels and from other proton-sensing GPCRs with distinct pH sensitivities and signaling biases.

GPR68 is a proton-sensing Class A GPCR that becomes activated when extracellular pH shifts from physiological levels toward mild acidity. Biochemical and cellular studies show that GPR68 exhibits half-maximal activation around pH 6.8–7.0 and with progressively stronger activation as extracellular pH falls toward ~6.5, the range observed in acidic microdomains of human OA cartilage [13,15,24]. Protonation of key extracellular histidine residues drives conformational changes that engage intracellular signaling, making GPR68 a sensitive detector of the modest extracellular pH reductions typical of early and established OA [24]. Importantly, these activation properties are defined primarily through biochemical and cellular studies and do not, by themselves, establish a causal role for GPR68 in OA pathogenesis.

Upon activation by low pH, GPR68 couples to several signaling pathways relevant to joint cell behavior. The dominant response in many systems is Gq/11-PLC activation, leading to inositol phosphate generation, intracellular Ca^2+^ release, and downstream ERK1/2 activation [24,25,28]. GPR68 can also stimulate cAMP/PKA-CREB signaling and activate G12/13-RhoA/ROCK pathways, linking extracellular acidity to cytoskeletal remodeling, cell contractility, and mechanotransduction [29,30]. Most mechanistic characterization of these pathways has been performed in non-joint tissues, including airway smooth muscle, intestinal epithelium, endothelial cells, and cancer models, and their engagement in OA joint cells remains incompletely defined. Collectively, these pathways—MAPK, CREB, NF-κB, and RhoA/ROCK—control many inflammatory, catabolic, and matrix-regulatory genes that are dysregulated in OA. Thus, their relevance to OA is inferred from pathway convergence rather than direct demonstration in joint tissues.

Although GPR68 can also respond to mechanical stimuli, current evidence suggests that mechanosensitivity is largely acid-dependent, functioning more as an amplifier of proton-driven activation rather than an independent pure mechanoreceptor [30,31,32]. Accordingly, mechanotransduction should be considered a modulatory feature of GPR68 signaling rather than its primary mode of activation in the context of OA. Taken together, the activation pH range of GPR68, its intracellular signaling repertoire, and its expression profile in human OA cartilage support its consideration as a proton-responsive receptor in the acidic joint microenvironment, while recognizing that functional roles in OA require further direct experimental validation.

## 3. GPR68 Expression in OA-Relevant Joint Compartments

**Figure 3:**
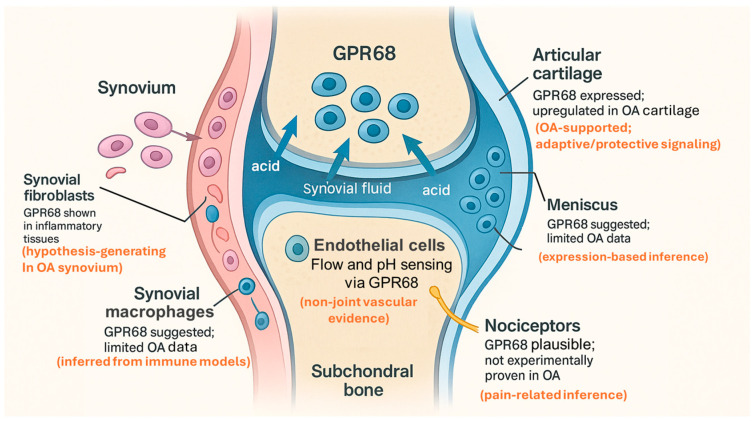
Anatomical distribution of GPR68 expression in OA-relevant joint tissues. Schematic cross-section of the human knee joint illustrating GPR68 expression in cartilage (chondrocytes), synovial lining (fibroblast-like synoviocytes and macrophages), meniscus (fibrochondrocytes), subchondral bone (osteoblast/osteoclast lineage), and endothelial cells, with possible expression in nociceptive nerve fibers. The diagram highlights the compartment-specific contexts in which acidic microdomains may engage GPR68 during OA progression. This compartmental schematic summarizes tissue-specific roles of GPR68 in the OA joint. Experimental support is strongest in articular cartilage, whereas roles in synovium, endothelium, and sensory neurons are based largely on non-joint disease models and are presented as hypothesis-generating. The figure integrates context-dependent signaling outputs of GPR68 rather than implying uniform receptor function across joint tissues.

Available data suggest that GPR68 is not restricted to cartilage but is expressed in multiple cell types that participate in OA pathology, including chondrocytes, synovial fibroblasts and macrophages, endothelial cells, and, at least in some contexts, neurons. However, the strength of evidence supporting GPR68 expression and function varies substantially across joint compartments, with the most direct OA-specific data available for articular cartilage. Most mechanistic work has been carried out in non-OA systems such as intestine, lung, bone, and cancer models, but collectively these studies provide a framework for how GPR68 could integrate acidic and inflammatory cues across joint compartments [14,24,31,33,34]. Accordingly, evidence outside cartilage should be interpreted as hypothesis-generating rather than definitive for OA. Figure 3 provides an anatomical overview of GPR68 expression across OA-relevant joint compartments, based on current evidence from cartilage, synovium, meniscus, bone, vasculature, and sensory nerves (Figure 3).

### 3.1. Chondrocytes and Bone Cells

In articular cartilage, chondrocytes are constantly exposed to mechanical loading and modest fluctuations in extracellular pH, which become more pronounced in OA joints. Proton-sensing GPCRs, including GPR68, are expressed in cartilage and bone cells and are activated in the mildly acidic range relevant for degenerative disease [24,31]. In human OA cartilage, GPR68 is upregulated compared to healthy controls. We find that upregulation of GPR68 is not a pathological response of chondrocytes, but rather a protective response-a molecular attempt by the cell to sense the acidic, inflammatory environment and activate compensatory mechanisms to restrain catabolic damage. Therefore, increased GPR68 expression in OA cartilage is considered as a microenvironment-responsive or adaptive change and does not, by itself, imply a pathogenic role in cartilage degeneration. We next showed that pharmacologic activation of GPR68 inhibits IL1β induced MMP13 expression in human chondrocytes, indicating that GPR68 signaling may exert protective, anti-catabolic effect under acidic and inflammatory conditions [26,33].

In osteoblast and osteoclast lineage cells, GPR68 is required for sensing low pH and contributes to acid-induced changes in bone turnover, including osteoclastogenesis and bone resorption [33,35,36]. Although these findings are not specific to OA, they support the broader concept that GPR68 can translate acidic microenvironments into transcriptional responses in skeletal cells.

### 3.2. Synovial Fibroblasts and Macrophages

Direct evidence for GPR68 expression or function in OA synovium is currently limited. However, several lines of evidence from other tissues show that GPR68 is expressed in fibroblasts and myeloid cells and is responsive to acidic, inflammatory microenvironments. In intestinal disease models, GPR68 is upregulated in inflamed and fibrotic tissue, with expression detected in stromal fibroblasts, myofibroblasts, and infiltrating immune cells [37,38,39]. Genetic deletion of GPR68 in mice or pharmacologic inhibition reduces colitis severity and diminishes inflammatory cytokines, chemokines, and collagen deposition, indicating that GPR68 contributes to macrophage recruitment, fibroblast activation, and tissue fibrosis under chronic inflammatory and acidic conditions [37,38,39].

Acidosis-induced GPR68 signaling in primary fibroblasts has been shown to drive inositol phosphate production, RhoA activation, and cytoskeletal remodeling [33]. In macrophages and other myeloid cells, GPR68 deficiency reduces inflammatory responses and immune cell recruitment in non-joint inflammatory models [33,37,38,40].

Together, these observations suggest that GPR68 could modulate synovial fibroblast and macrophage behavior in an acidified OA synovium; however, this interpretation remains inferential and should be regarded as hypothesis-generating until validated directly in OA tissues.

### 3.3. Endothelium and Subchondral Microvasculature

Endothelial cells in and around joints are exposed to both inflammatory mediators and altered mechanical forces, particularly in subchondral bone and peri-synovial microvessels. GPR68 is robustly expressed in arteriolar endothelial cells and functions as a flow sensor that integrates shear stress with extracellular pH, regulating vasodilation and vascular remodeling [30]. In mice, loss of GPR68 in the endothelium impairs flow-mediated dilation and leads to defects in vascular physiology, establishing GPR68 as a bona fide mechanosensitive, proton-sensing GPCR in the vascular wall [30].Transcriptomic studies indicate that GPR68 participates in acid-responsive endothelial gene programs [31,33,38,39,41]. However, joint-specific endothelial studies in OA are lacking, and any proposed role for GPR68 in synovial angiogenesis or immune cell trafficking should be considered mechanistic plausibility rather than demonstrated OA biology.

### 3.4. Sensory Neurons and Nociception

Pain is a dominant clinical feature of OA, and joint acidosis has long been recognized as a potent driver of nociceptor activation, primarily via ion channels such as ASICs and TRPV1. Compared with these channels, there is much less direct in vivo evidence for GPR68 in articular pain pathways. Proton-sensing GPCRs, including GPR68, are expressed in various neuronal populations and can modulate neuronal excitability in response to extracellular pH changes, as summarized in recent reviews [31,34,40].

Although GPR68 has been proposed to function as a coincidence detector of extracellular protons and mechanical stimuli [32], its contribution to OA pain remains unproven and substantially less supported than that of ASIC3 and TRPV1. Thus, GPR68 should be viewed as a plausible but unvalidated modulator of acid-mechanical integration in OA nociception. Together, these findings show that GPR68 is present across multiple joint compartments, although the strength and type of evidence vary substantially between tissues. Table 1 summarizes the current evidence supporting GPR68 expression across joint tissues, highlighting where data are strong, moderate, or limited.

## 4. Acid-Driven Signaling Through GPR68: Mechanisms and OA-Relevant Implications

GPR68 belongs to the small family of proton-sensing GPCRs that are activated when extracellular pH drops into the mildly acidic range (approximately pH 6.4–7.0) characteristic of inflamed or ischemic tissues [24,46]. Histidine residues in the extracellular regions and within the transmembrane bundle confer proton sensitivity, allowing the receptor to translate modest changes in pHe into conformational states that engage multiple G-protein and β-arrestin pathways [31]. Although most mechanistic work has been performed outside the joint, these studies define a consistent signaling “toolkit” that can be mapped onto OA biology: Gq/11-PLC-Ca^2+^-PKC-MAPK, Gs/Gq-cAMP-PKA-CREB, G12/13-RhoA-ROCK-YAP/TAZ, and β-arrestin-biased ERK and stress-kinase pathways [28,29,32,37,46,47]. These pathways are therefore discussed as mechanistic frameworks rather than OA-proven signaling events.

### 4.1. Core Signaling Logic from Non-Joint Tissues

Early work in heterologous systems established GPR68 as a proton-activated receptor that robustly stimulates inositol phosphate production, consistent with Gq/11-PLC activation [24]. Pharmacological studies using sphingosylphosphorylcholine as a tool ligand showed that receptor activation increases both inositol phosphates and cAMP, indicating coupling to Gq/11 and Gs in a pH-dependent manner [29]. In airway smooth muscle cells, small decreases in extracellular pH (from 7.4 to 7.0–6.8) activate GPR68 and trigger Ca^2+^ mobilization, ERK1/2 and p38 MAPK phosphorylation, and contraction, confirming that proton-evoked GPR68 signaling can integrate Ca^2+^, MAPK, and contractile responses in primary cells [28].

Downstream of these canonical G-protein pathways, RhoA-ROCK and Hippo-YAP signaling have emerged as important mediators of proton-sensing GPCR responses. In endothelial and mesenchymal cells exposed to mild acidosis, proton-sensing GPCRs signal through G12/13 and Rho GTPases to activate YAP, thereby promoting cell proliferation and survival [47]. This GPCR-G12/13-Rho-YAP axis is likely relevant to GPR68, which shares similar coupling and can activate RhoA and mechanotransduction pathways in vascular cells [30,31]. However, direct activation of this pathway by GPR68 has not yet been demonstrated in joint-resident cells.

In the intestinal epithelium and lamina propria, GPR68 expression and activity increase in the context of chronic inflammation and local acidification. In models of inflammatory bowel disease, GPR68 is upregulated in epithelial cells and myeloid populations, and receptor activation modulates barrier function, cytokine production, and fibrotic remodeling [37,38,39]. Functional studies in intestinal epithelial models show that acid-driven GPR68 activation induces ER stress via the IRE1α-JNK pathway and regulates autophagy-related responses, linking extracellular acidosis to the unfolded protein response and stress-kinase activation [42]. These observations provide mechanistic insight but are derived from non-joint tissues.

**Figure 4:**
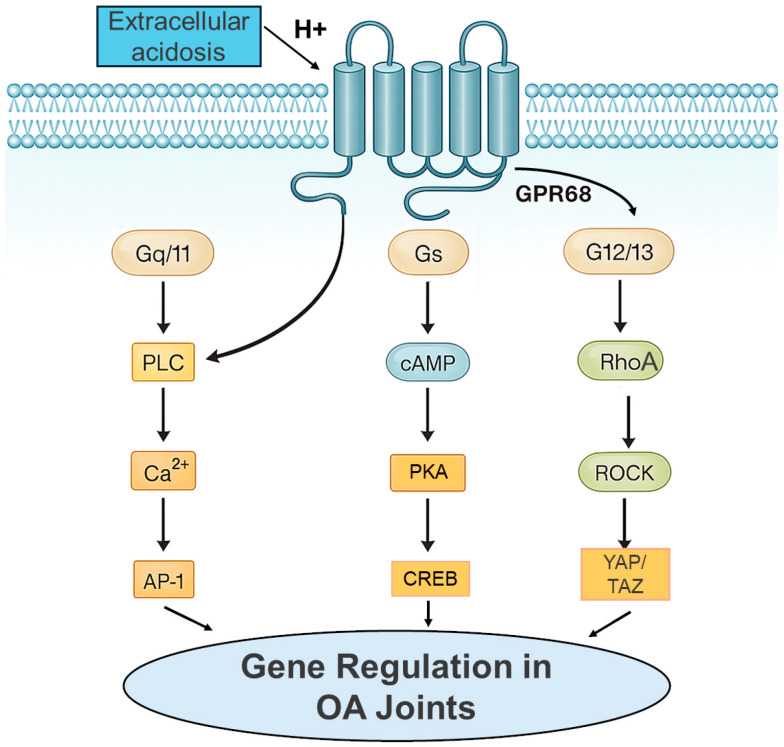
Acid-activated GPR68 signaling pathways relevant to OA. Extracellular acidosis activates GPR68 and engages Gq/11-PLC-Ca^2+^-PKC-MAPK, Gs-cAMP-PKA-CREB and G12/13-RhoA-ROCK-YAP/TAZ modules, which converge on transcription factors such as AP-1, NF-κB, CREB, and YAP/TAZ to regulate OA-relevant gene programs including matrix-degrading enzymes, inflammatory cytokines and chemokines, COX-2/PGE_2_ in joint cells. The signaling pathways depicted represent canonical GPR68 coupling described across multiple cell types and tissues. Their relative engagement and downstream effects in OA joint cells are likely context dependent, influenced by extracellular pH, cell type, receptor expression level, and microenvironmental cues.

Collectively, these non-joint data indicate that proton-dependent GPR68 activation is not restricted to a single G-protein but instead recruits a network of signaling modules: PLC-IP_3_-Ca^2+^ and PKC, ERK/p38/JNK MAPKs, cAMP-PKA-CREB, RhoA-ROCK-YAP, and ER-stress pathways. Figure 2 summarizes these acid-activated GPR68 signaling pathways, highlighting the convergence of Gq/11, Gs, and G12/13 modules on transcriptional hubs relevant to OA (Figure 4). Integrative reviews of proton-activated GPCRs reinforce this view and highlight GPR68 as a pleiotropic pH sensor whose signaling profile is strongly influenced by cell type, pH range, receptor expression level, and co-receptor context [33,46].

### 4.2. Relevance to OA Gene Programs

Many of the downstream pathways engaged by GPR68 in non-joint tissues map directly onto transcriptional programs that are central to OA. In chondrocytes, synoviocytes, and osteoblasts, Gq/11-PLC-Ca^2+^-PKC and ERK/p38 MAPKs drive AP-1-dependent expression of catabolic enzymes such as MMP13 and ADAMTS5, as well as COX-2 and prostaglandin E_2_, in response to inflammatory cytokines and mechanical stress [48,49,50]. These OA-relevant transcriptional programs are well established; however, direct evidence that GPR68 is the upstream driver of these pathways in OA joint cells remains limited. These same pathways are activated downstream of proton-sensing GPCRs, including GPR68, in bone and intestinal models, where GPR68-dependent Ca^2+^ and MAPK signaling regulate COX-2, prostaglandins, and inflammatory genes [35,38,42,51]. Accordingly, the OA interpretation here should be considered mechanistic plausibility based on pathway convergence rather than OA-proven receptor signaling.

The cAMP-PKA-CREB arm is also relevant for OA-associated cytokine and chemokine production. In cancer-associated fibroblasts, GPR68 senses the acidic tumor microenvironment and enhances IL-6 expression via a cAMP-PKA-CREB pathway, thereby promoting tumor cell proliferation [43]. IL-6 and related chemokines (for example, CCL2, CXCL8/IL-8) are key mediators of synovial inflammation and cartilage matrix breakdown in OA; it is therefore plausible that similar GPR68-cAMP-CREB signaling modules operate in acidic joint niches, even though this has not yet been directly demonstrated. We therefore present this pathway as hypothesis-generating for OA synovium and cartilage until joint-resident cell validation is available.

RhoA-ROCK-YAP signaling provides another link between proton-sensing GPCRs and OA-relevant phenotypes. In endothelial and mesenchymal cells, reduced pHe activates YAP through G12/13-RhoA, promoting proliferation, survival, and fibroblast activation [47,52]. YAP/TAZ activity in chondrocytes and synovial fibroblasts is known to modulate matrix production, fibrosis, and mechanosensitive gene programs in experimental OA models, making this axis a particularly attractive candidate for mediating acid-mechanical crosstalk downstream of GPR68. However, direct demonstration of GPR68→RhoA/ROCK→YAP/TAZ signaling in OA joint cell types is currently lacking, and this axis should be interpreted as a plausible mechanistic bridge rather than established OA signaling.

Finally, ER-stress and unfolded protein response pathways are increasingly recognized in OA cartilage and synovium. The observation that GPR68 activation in intestinal epithelial cells induces ER stress via IRE1α-JNK [42] provides a mechanistic template by which chronic acidosis in the joint could contribute to maladaptive stress responses, chondrocyte dysfunction, and senescence. This remains an extrapolation from non-joint systems, and joint-specific testing is needed to determine whether GPR68 engages ER-stress pathways in cartilage or synovium during OA.

### 4.3. OA-Relevant Indirect Evidence

Direct studies of GPR68 signaling in OA joint cells are still sparse but work on skeletal and disk tissues offers relevant clues. In bone cells, metabolic acidosis increases intracellular Ca^2+^ via GPR68 and modulates osteoclast and osteoblast function, implicating the receptor in bone remodeling under acidic conditions [36,51]. In rat intervertebral disk endplate chondrocytes, GPR68 is the predominant proton-sensing GPCR, is strongly upregulated by extracellular acidosis, and mediates acid-induced apoptosis through Ca^2+^-dependent activation of calpain, calcineurin, and downstream apoptotic effectors [53]. These findings show that GPR68 can couple extracellular acidosis to Ca^2+^-sensitive proteases and cell-death pathways in cartilage-related tissues, but these observations derive from disk/endplate contexts and should not be assumed to operate identically in articular cartilage. Notably, in human articular chondrocytes we previously reported that pharmacologic activation of GPR68 suppresses IL1β-induced MMP13 under acidic conditions [26], supporting the possibility of context-dependent and potentially protective outputs in cartilage rather than uniform pro-death signaling.

GPR68 has also been implicated in acid-driven fibroblast activation and fibrosis outside the joint. In experimental colitis, intestinal activation of GPR68 promotes fibrogenesis, and pharmacological inhibition attenuates inflammatory and fibrotic responses [37,39]. These data align with the concept that acid-sensing GPCRs influence stromal cell phenotypes and extracellular matrix remodeling, both key features of synovial fibrosis in OA. However, direct evidence linking GPR68 to synovial fibrosis in OA is currently lacking, and this linkage should be considered hypothesis-generating.

Taken together with the cartilage expression data and emerging OA models using pharmacologic GPR68 modulators, the non-joint literature supports a coherent picture: in acidic microenvironments, GPR68 coordinates Ca^2+^, MAPK, cAMP, RhoA-ROCK, ER-stress, and YAP/TAZ signaling modules that are already known to regulate matrix catabolism, inflammatory mediator production, cell survival/apoptosis, and fibroblast activation in OA tissues. Here, “coherent picture” refers to mechanistic plausibility based on pathway overlap, not to direct OA validation of GPR68 signaling across joint compartments. The main gap is not the plausibility of these pathways in OA but the lack of direct, cell-type-specific mechanistic studies in joint cells.

In summary, acid-driven GPR68 signaling is mechanistically well defined in non-joint tissues: mild extracellular acidosis engages multi-branch G-protein and β-arrestin pathways that converge on AP-1, NF-κB, CREB, ER-stress, and YAP/TAZ transcriptional programs. These same hubs regulate key OA genes, including matrix-degrading enzymes, inflammatory cytokines and chemokines, COX-2/PGE_2_, and fibrosis-related effectors. The available evidence from bone, intervertebral disk, intestinal, airway, and tumor models supports a working model in which GPR68 functions as a proton-sensing hub capable of influencing OA-relevant gene programs when joint pHe falls into the acidic range. However, rigorous in vitro and in vivo studies in defined joint cell populations are still needed to convert this mechanistic plausibility into direct evidence in OA.

## 5. GPR68-Responsive Signaling and OA Gene Programs

Joint acidosis contributes to a broad spectrum of transcriptional responses that underlie cartilage degeneration and synovial inflammation in OA. Although direct studies examining acid-driven GPR68 signaling in articular chondrocytes, synoviocytes, or synovial macrophages are still lacking, substantial mechanistic information from other tissues shows that proton-dependent GPR68 activation engages signaling pathways-ERK1/2, p38, NF-κB, CREB, RhoA-ROCK, YAP/TAZ, and IRE1α-JNK that tightly regulate the major gene programs implicated in OA. Throughout this section, the involvement of GPR68 in OA gene regulation is discussed as mechanistic inference based on pathway convergence rather than as direct evidence of receptor-driven signaling in joint tissues. These conserved pathways provide a coherent framework for understanding how extracellular acidosis, acting through GPR68, may shape inflammatory and catabolic gene expression in the OA joint.

### 5.1. Catabolic Matrix Genes (MMP13, MMP3, ADAMTS4/5)

Matrix metalloproteinases (MMP13, MMP3) and aggrecanases (ADAMTS4/5) are central effectors of cartilage extracellular matrix loss in OA. Their expression in human chondrocytes is strongly regulated by ERK1/2, p38 MAPK, PKC, and AP-1 transcription factors [48,49,50]. Acidosis is known to activate these same pathways through GPR68 in non-joint systems: mild decreases in pHe stimulate Gq/11-PLC-Ca^2+^ signaling and MAPK activation in airway smooth muscle and intestinal epithelial cells [28,42]. These findings indicate that acidic activation of GPR68 can engage canonical catabolic signaling cascades. However, direct demonstration that GPR68 activates these catabolic pathways in OA chondrocytes is currently lacking.

In our earlier work, we found that GPR68 expression is increased in human OA cartilage and aligns with regions showing matrix-degrading activity, and that pharmacologic activation of GPR68 suppresses MMP13 expression in human chondrocytes exposed to inflammatory and mildly acidic conditions [26]. These findings suggest that GPR68 upregulation in diseased cartilage may represent an adaptive, anti-catabolic response rather than a driver of matrix breakdown. This observation underscores the context-dependent nature of GPR68 signaling and cautions against assuming uniform catabolic outputs downstream of receptor activation.

### 5.2. Inflammatory Cytokines and Chemokines (IL-6, IL-8, CCL2)

Synovial inflammation in OA is marked by elevated production of IL-6, IL-8 (CXCL8), and the monocyte chemoattractant CCL2, all of which contribute to macrophage recruitment, synovial hyperplasia, and cartilage catabolism. These genes are controlled by NF-κB, ERK/p38, and cAMP-CREB pathways in synoviocytes and chondrocytes [48,54].

GPR68 activation drives similar inflammatory outputs in acidic inflammatory environments outside the joint. In intestinal inflammation, GPR68 increases cytokine and chemokine expression in epithelial and stromal cells [38,39]. In cancer-associated fibroblasts, acidic activation of GPR68 promotes IL-6 expression via a cAMP-PKA-CREB pathway [43]. Proton-sensing GPCRs broadly enhance NF-κB and MAPK signaling across inflammatory cell types [46]. Given the established role of these pathways in OA synovitis, it is plausible that GPR68 may influence inflammatory gene programs in acidified joint niches. At present, this remains a hypothesis-generating interpretation, as direct evidence for GPR68-dependent cytokine regulation in OA synovium or cartilage is limited.

### 5.3. COX-2, PGE_2_, and Eicosanoid Signaling

COX-2 (PTGS2) and its downstream mediator PGE_2_ are key inflammatory effectors in OA cartilage and synovium, promoting matrix breakdown and sensitizing nociceptors. COX-2 expression is induced by cytokines, mechanical load, and acidic pH in joint tissues [55,56]. Acid-driven activation of GPR68 increases COX-2 expression and prostaglandin production in smooth muscle and intestinal epithelial models [28,38,42]. Because COX-2/PGE_2_ production is enhanced in OA, these mechanistic data from non-joint systems suggest that GPR68 could contribute to prostaglandin-mediated inflammation during joint acidosis. However, direct evidence that GPR68 drives COX-2/PGE_2_ induction in OA joint-resident cells is currently limited, and this linkage should be considered hypothesis-generating rather than established OA mechanism.

### 5.4. RhoA–ROCK, YAP/TAZ, and Synovial Fibroblast Activation

Fibroblast-like synoviocytes undergo phenotypic transformation in OA, characterized by cytoskeletal remodeling, increased contractility, and matrix deposition. These processes depend on RhoA-ROCK signaling and YAP/TAZ transcriptional activity. Proton-sensing GPCRs trigger G12/13-RhoA-ROCK-YAP pathways in mesenchymal cells exposed to mild acidosis [47,52]. GPR68 itself has been shown to activate RhoA in endothelial and stromal cells and participates in mechanosensitive signaling [30]. These parallels suggest that acid-dependent activation of GPR68 may contribute to fibroblast activation and synovial remodeling, although direct evidence in human OA synoviocytes is still needed. Accordingly, any proposed role for GPR68 in OA synovial fibroblast activation should be interpreted as mechanistic plausibility based on non-joint systems rather than direct OA validation. In addition, given the context-dependent outputs reported for GPR68 across tissues, the directionality of GPR68 effects in synovium (pro- versus anti-inflammatory or pro- versus anti-fibrotic) remains unresolved and requires direct OA-focused testing.

### 5.5. ER-Stress, Ca^2+^ Signaling, and Chondrocyte Survival

ER stress and the unfolded protein response are increasingly recognized in OA cartilage, where they contribute to chondrocyte dysfunction, apoptosis, and senescence. Acid activation of GPR68 induces ER stress through the IRE1α-JNK pathway in epithelial cells under inflammatory acidic conditions [42]. In addition, GPR68 mediates acid-induced Ca^2+^ influx and apoptosis in intervertebral disk endplate chondrocytes [53], demonstrating that proton-sensing GPCRs can couple acidosis to stress and cell-death pathways in cartilage-related tissues. These data provide a mechanistic template for considering GPR68 as a contributor to acid-driven chondrocyte injury in OA. However, these ER-stress and apoptosis mechanisms are derived from non-articular systems and should not be assumed to operate identically in articular chondrocytes during OA. Notably, we previously reported that pharmacologic activation of GPR68 suppresses IL1β-induced MMP13 expression in human articular chondrocytes under acidic conditions [16], indicating that GPR68 signaling can also support adaptive or protective outputs depending on cellular context.

Overall, although direct mechanistic studies of GPR68 signaling in joint cells are limited, acid-dependent activation of GPR68 in other tissues engages signaling modules that are central to OA pathobiology, including MAPK/AP-1, NF-κB, CREB, RhoA-ROCK-YAP/TAZ, and ER-stress pathways. These pathways regulate the expression of matrix-degrading enzymes, inflammatory cytokines and chemokines, prostaglandin synthesis, fibroblast activation, and cell-stress responses-core components of OA gene programs. Together with evidence of GPR68 upregulation in human OA cartilage, these findings support a potential role for GPR68 as a proton-responsive signaling receptor in acidic joint microenvironments and highlight the need for OA-focused mechanistic studies.

Collectively, these observations indicate that GPR68 does not exert a uniform biological effect across tissues, but instead functions as a context-dependent interpreter of extracellular acidity. Differences in GPR68 signaling outcomes across systems likely reflect variation in receptor density, local pH magnitude and duration, extracellular matrix stiffness, and the relative engagement of Gq/11-, Gs-, and G12/13-mediated pathways. In environments characterized by chronic inflammation or tumor-associated acidosis, sustained GPR68 activation may preferentially engage pro-inflammatory or pro-survival programs, whereas in mesenchymal or stromal contexts, biased coupling toward cAMP/CREB or anti-fibrotic pathways has been observed. In cartilage, where extracellular pH reductions are modest and spatially restricted, GPR68 activation may instead support adaptive or protective transcriptional responses, consistent with suppression of IL1β-induced MMP13 expression in human chondrocytes. By contrast, in synovial tissues exposed to persistent inflammation, immune cell infiltration, and altered matrix mechanics, GPR68 signaling could plausibly reinforce inflammatory or chemotactic programs. Thus, apparent differences in GPR68 function across tissues are best viewed not as inconsistencies, but as manifestations of context-specific signaling integration.

## 6. Therapeutic Implications of Targeting GPR68 in OA

Evidence that GPR68 acts as a proton sensor in multiple inflammatory and degenerative settings has led to serious consideration of this receptor as a drug target [25,33,40]. In OA, joint acidosis is increasingly recognized as a disease-relevant feature, and GPR68 is upregulated in human OA cartilage and associated with matrix-degrading signatures [26,40]. These observations raise two broad therapeutic questions: (i) whether GPR68 should be pharmacologically activated or inhibited in OA, and (ii) how such modulators could be delivered and monitored in a pH-heterogeneous joint environment. Because GPR68 signaling is highly context dependent, with both pro-inflammatory and tissue-protective effects reported in different organs [25,33,40], any strategy for OA will likely require careful attention to cell type, disease stage, and route of administration rather than a simple “on/off” approach. Importantly, to date, available small-molecule GPR68 modulators have not yet been evaluated in preclinical OA models, and therapeutic implications in OA should therefore be considered hypothesis-generating.

### 6.1. Pharmacologic Modulation of GPR68 via Agonists, PAMs, and Inhibitors

The pharmacology of GPR68 has advanced rapidly over the last decade. The first small-molecule positive allosteric modulator (PAM), Ogerin, was identified in a screen for GPR68 modulators and shown to potentiate proton-induced Gs–cAMP signaling [57]. Subsequent structure-activity work produced improved Ogerin analogs with higher potency and selectivity, enabling in vitro and in vivo studies of GPR68 biology [57]. Functionally, Ogerin has been used to enhance GPR68 signaling in several models: it suppresses TGFβ1-induced myofibroblast differentiation and promotes reversion of mature myofibroblasts, suggesting an anti-fibrotic profile in lung and tendon-associated fibroblasts [58]. These data highlight that enhancing GPR68 activity in some mesenchymal compartments can dampen fibrotic and contractile responses. Importantly, these effects have been demonstrated exclusively in non-joint systems and have not yet been evaluated in OA models.

In parallel, an unbiased developmental screen in zebrafish led to the discovery of Ogremorphin, a first-in-class small-molecule GPR68 inhibitor [59,60]. Ogremorphin has been shown to block GPR68-dependent migration and metastasis of cancer cells, and more recent work indicates that GPR68 inhibition using Ogremorphin or related compounds can induce ferroptosis and radiosensitization in glioblastoma and other tumor models [59,60,61]. In airway epithelium, Ogremorphin suppresses GPR68-dependent MUC5AC expression, pointing to an anti-secretory, anti-inflammatory effect in mucosal tissues [59,60]. Additional Ogremorphin-based inhibitors are being explored for barrier-protective and anti-inflammatory effects in the pulmonary vasculature [62]. To date, none of these inhibitory strategies has been tested in the context of OA or in joint-resident cell populations.

Taken together, these studies show that both activation and inhibition of GPR68 are pharmacologically feasible and can yield beneficial outcomes, but in very different disease contexts. Ogerin-like PAMs may be advantageous where GPR68 signaling constrains fibrosis or supports adaptive stress responses, whereas Ogremorphin-like antagonists may be more appropriate where GPR68 promotes persistent inflammation, cell survival, or malignancy [25,40]. For OA, however, the directionality of therapeutic modulation (activation versus inhibition) remains unresolved and is likely to be highly cell-type- and microenvironment-dependent. For OA, where GPR68 is expressed in chondrocytes, synovial fibroblasts, macrophages, endothelial cells, and sensory neurons, it is almost certain that the net effect of either class of modulator will depend on compartment-specific roles of GPR68 that are not yet fully defined. Accordingly, any therapeutic implications for OA should be regarded as conceptual and hypothesis-generating rather than evidence-based at present. A comparative summary of emerging therapeutic strategies for modulating GPR68 in OA, including agonists, inhibitors, and delivery platforms, is provided in Table 2.

### 6.2. Lessons from Non-Joint Disease Models

Several in vivo studies outside the joint provide important guidance for OA drug development. In glioblastoma, GPR68 supports a prosurvival ATF4 pathway; Ogremorphin-mediated inhibition drives lipid peroxidation, iron-dependent cell death, and sensitization to radiotherapy [59,60,61]. In the vasculature, GPR68 is essential for flow sensing and vascular tone, indicating that systemic inhibition could affect endothelial function and blood pressure [30]. Conversely, Ogerin-mediated enhancement of GPR68 signaling has shown protective effects by limiting myofibroblast differentiation and fibrosis in models of lung and tendon injury [58]. Reviews synthesizing these data emphasize that GPR68 behaves as a context-dependent pH sensor, with both pro- and anti-inflammatory influences depending on the tissue, cell type, and balance of Gq, Gs, G12/13, and β-arrestin signaling [25,33,40]. Importantly, all of these mechanistic and therapeutic insights are derived from non-joint disease models and have not yet been directly tested in OA.

For OA, this implies that cell-specific targeting will likely be crucial. Enhancing GPR68 signaling in chondrocytes might support adaptive responses to acidosis, whereas inhibiting GPR68 in synovial fibroblasts or macrophages could reduce inflammatory and chemotactic programs. These proposed strategies remain hypothesis-generating and are based on extrapolation from non-joint systems rather than direct OA evidence. Conversely, systemic GPR68 inhibition may have unintended consequences for vascular physiology or host defense. These considerations argue strongly for local, intra-articular delivery and for preclinical work in cell-type-specific genetic models to define whether chondrocyte, synovial fibroblast, macrophage, or sensory-neuron GPR68 should be the primary therapeutic focus in OA. At present, such cell-type–resolved validation in OA models is lacking.

### 6.3. Local and pH-Responsive Intra-Articular Delivery

Intra-articular drug delivery is an attractive route for modulating GPR68, as it allows high local concentrations with limited systemic exposure. A growing body of work has explored injectable hydrogels, nanoparticles, and liposomes for OA therapy, including systems that are responsive to pH, enzymes, or mechanical loading [64,65,68]. pH-responsive carriers are particularly relevant for a proton-sensing target such as GPR68. Hyaluronic acid-modified metal–organic framework nanoparticles have been used to deliver anti-inflammatory drugs selectively to inflamed, acidic cartilage and synovium, reducing OA progression in vivo [66]. More recently, injectable pH-responsive and cartilage-targeted liposomal systems have been developed to release cargo preferentially in low-pH OA joints [67]. Reviews of responsive hydrogels for OA highlight the potential of combining pH-sensitive matrices with disease-relevant triggers to achieve flare-responsive or microenvironment-specific release [63,65].

These platforms could, in principle, be adapted to deliver small-molecule GPR68 modulators directly into the joint. A GPR68 agonist or PAM encapsulated within a pH-responsive hydrogel might be designed to release primarily in cartilage regions where pHe drops into the receptor’s activation window (pH ~6.5–7.0), whereas an antagonist formulation might be optimized to target synovium or inflamed pannus tissue. Co-delivery with conventional anti-inflammatory drugs or senolytics is another possibility. These delivery concepts are currently theoretical with respect to GPR68 and are extrapolated from OA delivery platforms developed for other therapeutic agents. However, no studies to date have combined GPR68 modulators with such OA-specific delivery systems, and this remains a clear area for translational development. Accordingly, proposed delivery strategies for GPR68 in OA should be regarded as hypothesis-generating rather than validated approaches.

### 6.4. pH Imaging and Pharmacodynamic Readouts

A major challenge for any pH-sensing receptor therapy is how to monitor target engagement in vivo. AcidoCEST-UTE MRI has emerged as a promising non-invasive method to quantify extracellular pH in cartilage, meniscus, and joint fluid in humans [13,15,69]. Using intra-articular administration of iodinated contrast agents, these studies showed that cartilage and meniscus pHe can be measured reproducibly on clinical scanners, and that patients with knee OA have significantly lower pHe in cartilage, meniscus, and synovial fluid than individuals without OA [13,15]. Lower pHe correlated with worse pain and KOOS scores, supporting the functional relevance of acidification in symptomatic disease [15].

If GPR68-targeted therapies move toward clinical testing, acidoCEST-UTE MRI could be used to stratify patients with acidic joints, to monitor changes in pHe with treatment, and to link pH modulation to molecular and clinical outcomes. At present, this application remains conceptual, as no GPR68-directed therapies have been evaluated in OA clinical or preclinical studies. In parallel, pharmacodynamic gene signatures-such as changes in GPR68-responsive cytokines, chemokines, or matrix genes in synovial fluid cells or cartilage biopsies-could serve as complementary readouts. However, such molecular signatures have not yet been validated as specific indicators of GPR68 pathway engagement in OA. Integration of pH imaging with transcriptional profiling would allow identification of low-pHe OA endotypes that exhibit joint acidification within the GPR68 activation window. This endotype-based framework is hypothesis-generating and will require prospective validation. In this context, acidoCEST-UTE MRI provides a non-invasive pharmacodynamic readout by directly reporting changes in extracellular pHe within the activation range of GPR68 following therapeutic intervention.

### 6.5. Outstanding Challenges and Future Directions

Despite clear conceptual appeal, several challenges need to be addressed before GPR68 can be considered a realistic OA drug target. First, cell-specific roles of GPR68 in chondrocytes, synovial fibroblasts, macrophages, endothelial cells, and sensory neurons in OA joints remain incompletely understood. Second, preclinical data using Ogerin or Ogremorphin have been generated mainly in cancer, brain, lung, and tendon models, and it is not yet known whether the same direction of modulation (activation versus inhibition) will be beneficial in OA. Third, systemic inhibition of GPR68 could affect vascular tone, barrier function, or host defense, arguing for intra-articular and possibly compartment-selective delivery. Importantly, no studies to date have directly tested GPR68 modulation in established OA disease models.

In the near term, a rational roadmap would involve: (i) defining cell-type-specific functions of GPR68 in OA using conditional genetic models; (ii) testing Ogerin-like PAMs and Ogremorphin-like inhibitors in surgically induced OA with local delivery; (iii) identifying pharmacodynamic markers and pH-imaging signatures that capture GPR68 pathway engagement; and (iv) integrating these data into a framework that can support endotype-guided, pH-aware clinical trials. At present, these steps remain prospective and hypothesis-driven rather than evidence-based. Such work would clarify whether GPR68 should be targeted as a protective sensor to be boosted or a pathogenic amplifier to be restrained in OA. Here, “protective” versus “pathogenic” framing is intended to reflect alternative mechanistic possibilities rather than established roles of GPR68 in OA.

## 7. Knowledge Gaps and Priorities for GPR68 Research in OA

Although substantial progress has been made in characterizing GPR68 as a proton-sensing receptor in inflammation, cancer, and stromal biology, several key gaps limit the translation of these findings into the OA field. Addressing these gaps is essential to determine whether GPR68 should be pursued as a therapeutic target in OA and whether its activity is beneficial, detrimental, or context dependent. Importantly, current evidence supporting a role for GPR68 in OA is strongest in articular cartilage, while proposed roles in other joint compartments remain less well defined.

### 7.1. Lack of Direct Mechanistic Evidence in Joint Cells

At present, no studies have directly examined acid-driven GPR68 signaling in primary human articular chondrocytes, fibroblast-like synoviocytes, or synovial macrophages. Most mechanistic insights come from intestinal epithelial cells [38,39,42], smooth muscle [28], endothelial cells [30], or cancer-associated fibroblasts [43]. These cells demonstrate strong GPR68 coupling to Gq/11-PLC-Ca^2+^, Gs-cAMP-CREB, G12/13-RhoA-ROCK, and ER-stress pathways, all of which regulate OA-relevant gene programs, but these observations derive from non-joint systems and cannot be assumed to operate identically in joint-resident cells. The degree to which these pathways are engaged in joint cells exposed to acidic microenvironments remains unknown. Establishing direct evidence in joint-derived cells is therefore a top priority for determining whether GPR68 signaling is functionally relevant in OA rather than inferred by pathway overlap.

### 7.2. Unclear Cell-Type-Specific Roles of GPR68 in the Joint

GPR68 is expressed in multiple joint compartments, including cartilage, synovium, bone cells, macrophages, endothelial cells, and nociceptive neurons [26,70]. It is unlikely that GPR68 performs the same function across these cell types. Examples highlighting this complexity include pro-inflammatory signaling downstream of GPR68 in intestinal epithelium [38], anti-fibrotic effects of GPR68 activation in stromal fibroblasts [58], and pro-survival ATF4 signaling in cancer cells [59,60,61]. Given these divergent roles, the net effect of GPR68 modulation in OA cannot be predicted without defining cell-specific contributions. Accordingly, whether GPR68 activation is protective, neutral, or detrimental is likely to be highly context dependent rather than uniform across the joint. Conditional genetic models targeting chondrocytes, synoviocytes, macrophages, or sensory neurons will be required to determine whether GPR68 activation is protective or pathogenic in each compartment and to avoid extrapolating conclusions from non-joint disease models.

### 7.3. Limited Understanding of Spatiotemporal pH Patterns in Human OA

Most OA studies measure synovial fluid pH, but extracellular pH varies across cartilage, meniscus, synovium, and subchondral bone. AcidoCEST-UTE MRI has shown that cartilage and meniscus are more acidic than synovial fluid, and that pHe varies spatially within the joint [13,15]. However, high-resolution pH maps have not been integrated with molecular profiling of joint tissues. Key questions include: which microdomains of cartilage or synovium fall within the activation window of GPR68 (pH ~6.5–7.0); whether acidic niches correlate with regions of inflammatory cell infiltration, matrix loss, or nociceptive innervation; and how pHe changes across early, moderate, and late OA. At present, these relationships remain undefined, limiting interpretation of how and where GPR68 signaling may be engaged in vivo. Defining these spatiotemporal patterns is essential for meaningful interpretation of GPR68 function in vivo.

### 7.4. Directionality of GPR68 Signaling in OA: Protective or Pathogenic?

GPR68 can exert divergent effects depending on cell type and microenvironmental conditions, but emerging data in OA point toward a protective role in cartilage. We previously showed that pharmacologic activation of GPR68 suppresses MMP13 expression in human articular chondrocytes, indicating an anti-catabolic, chondroprotective response [26]. This suggests that increased GPR68 expression in OA cartilage may reflect an adaptive attempt to counteract acid-driven matrix loss. This interpretation is supported by direct evidence in cartilage but should not be extrapolated to other joint compartments without validation.

Outside the joint, however, GPR68 exhibits both pro- and anti-inflammatory roles depending on tissue context. It promotes inflammatory and fibrotic pathways in intestinal epithelium and stroma during chronic inflammation [37,38,39], whereas in fibroblast populations, positive allosteric modulation of GPR68 with Ogerin reduces myofibroblast differentiation and fibrosis [58]. In cancer models, GPR68 activation can support survival pathways, while its inhibition enhances cell death and immune responsiveness [59,60,61]. These contrasting findings highlight that the dominant GPR68 output depends on the balance of Gq/PLC-Ca^2+^, cAMP-CREB, G12/13-RhoA-ROCK, and β-arrestin signaling engaged in each cell type.

In OA, the available evidence, particularly the suppressive effect of GPR68 activation on MMP13 supports a protection-oriented role in chondrocytes, whereas the functions of GPR68 in synovial fibroblasts, macrophages, endothelial cells, and neurons remain undefined. Determining whether GPR68 activation or inhibition is beneficial in these compartments will require direct experiments using cell-type-specific genetic models and pH-controlled stimulation in joint-derived cells. Overall, the current data suggest that GPR68 activation may be protective in cartilage, while its roles in other joint tissues remain unclear. This context dependence underscores the importance of cell-specific targeting rather than a uniform pharmacologic approach to modulating GPR68 in OA and reinforces that therapeutic directionality remains unresolved outside cartilage.

### 7.5. Absence of OA-Specific Preclinical Studies Using GPR68 Modulators

To date, neither Ogerin-like PAMs nor Ogremorphin-like inhibitors have been tested in rodent models of post-traumatic or spontaneous OA. All published functional studies of these compounds are in cancer, airway epithelium, vasculature, or fibrosis models [57,59,60,61,62]. Translating these molecules into OA research will require careful consideration of optimal intra-articular formulation, pH-responsive delivery strategies, dosing frequency and receptor desensitization, effects on synovial inflammation versus cartilage catabolism, and potential off-target systemic effects. Importantly, the absence of OA-specific preclinical testing represents a critical gap that currently limits any conclusions regarding therapeutic efficacy or safety in the joint. This remains a wide-open field with substantial opportunity for new discovery.

### 7.6. Lack of Pharmacodynamic Biomarkers for GPR68 Activity in OA

Reliable pharmacodynamic (PD) markers are essential for early-phase therapeutic development. Several GPR68-responsive pathways—MAPK/AP-1, NF-κB, CREB, COX-2/PGE_2_, CCL2, IL-6—are measurable in synovial fluid or synovial tissue, but none have been validated as specific markers of GPR68 engagement. pH-imaging technologies such as acidoCEST-UTE MRI offer a complementary biomarker strategy by mapping extracellular pH in vivo [13,15]. Integration of transcriptomic signatures with pH-imaging readouts could support the development of GPR68-specific PD assays to guide dose selection and patient enrichment. At present, however, such biomarkers remain indirect and require validation to establish specificity for GPR68 pathway activation in OA.

### 7.7. Need for Endotype-Guided Clinical Translation

OA is molecularly heterogeneous. Acidification, synovitis, macrophage infiltration, and fibrosis vary widely between patients. The success of any GPR68-targeted therapy will depend on identifying patients with low-pHe, GPR68-responsive OA endotypes. Stratification could be based on acidoCEST-UTE pH maps, synovial fluid biomarkers (for example, IL-6, CCL2, PGE_2_), transcriptomic signatures of proton-sensing pathways, or imaging markers of inflammation or fibrosis. Endotype-guided approaches are now standard in other inflammatory diseases and will be necessary for precision targeting of a receptor as context-sensitive as GPR68. Nevertheless, application of endotype-guided strategies to GPR68 in OA remains prospective and will require OA-specific validation.

## 8. Conclusions

Joint acidosis is increasingly recognized as an important and underappreciated component of OA pathophysiology. Across cartilage, synovium, and subchondral tissues, extracellular pH falls into a range that is sufficient to activate the proton-sensing receptor GPR68. However, the strongest direct evidence for GPR68 involvement in OA currently derives from articular cartilage, whereas roles in other joint compartments remain less well defined. Although direct mechanistic studies in human joint cells are limited, substantial evidence from epithelial, stromal, vascular, and cancer models demonstrates that GPR68 integrates mild decreases in pHe with G-protein signaling pathways that regulate inflammation, matrix turnover, cytoskeletal remodeling, and cell stress responses, all central features of OA biology. In human OA cartilage, GPR68 expression is increased and associated with transcriptional signatures of matrix degradation, suggesting that this receptor is responsive to disease-relevant microenvironmental cues rather than being a primary driver of pathology.

Taken together, these findings support a conceptual framework in which GPR68 acts as a molecular interpreter of acidic stress in the joint, translating changes in extracellular pH into gene programs that may shape inflammatory and catabolic activity. However, several essential questions remain unresolved, including the cell-type-specific roles of GPR68 in joint tissues, the beneficial or detrimental direction of receptor modulation, and the conditions under which GPR68 activation versus inhibition might improve disease outcomes. At present, no OA-specific disease models have directly tested pharmacologic modulation of GPR68. The development of pharmacologic modulators such as Ogerin-derived positive allosteric agonists and Ogremorphin-class inhibitors provides new tools to test these questions in vivo but their relevance to OA remains to be established.

Future progress will depend on combining accurate measurements of joint pHe with cell-specific genetic models, local intra-articular delivery strategies, and robust pharmacodynamic assays. These approaches will clarify how GPR68 operates within the acidified microenvironment of the OA joint and whether it can be leveraged as a therapeutic target. Importantly, any therapeutic or precision medicine application will require OA-specific validation and careful consideration of context-dependent effects across joint tissues. As the field moves forward, defining “low-pHe, GPR68-responsive OA endotypes” represents a hypothesis-generating framework that may enable pH-aware, precision medicine strategies aimed at modifying disease progression pending rigorous experimental and clinical validation.

## Figures and Tables

**Table 1:** Evidence matrix for GPR68 across joint-relevant tissues.

Joint Tissue/Cell Type	Evidence Type	Direction of Evidence	Strength of Evidence	Key References
Human OA cartilage	Bulk RNA-seq (public, GSE114007), qPCR (published), protein (limited)	Higher expression in OA cartilage; highest expressed proton-sensing GPCR among GPR4/65/68/132	Strong (OA-Specific)	[26,27]
Chondrocytes (in vitro)	qPCR and functional assays; MAPK and NF-κB engagement under acid stress	Acid-activated GPR68 signaling; modulation of inflammatory and catabolic transcripts	Moderate-Strong	[26,31,42]
Synovial fibroblasts (FLS)	Inferred from RNA-seq of inflamed FLS; functional parallels from non-joint fibroblasts	Possible upregulation under cytokine stimulation; acid-responsive pathways inferred from non-joint fibroblasts	Limited (OA-Specific)	[37,42]
Synovial macrophages	Evidence from macrophages in other inflamed tissues; GPR68-dependent inflammatory responses	Acid-enhanced macrophage activation; proton-dependent NF-κB sensitization, Hypothesized modulation of inflammatory programs	Limited, Moderate (non-OA)	[37,42]
Endothelial cells	Flow + pH co-sensing; mechanobiology experiments	Acid-enhanced GPR68 activation; endothelial inflammatory gene induction	Moderate (non-OA)	[43]
Bone cells (osteoblast/osteoclast lineage)	Acid-induced GPR68 signaling in osteoblasts; proton-dependent osteoclast regulation	Acid-sensing linked to bone remodeling pathways	Moderate (non-OA)	[44,45]
Sensory neurons	No direct OA evidence; indirect support from proton-sensing literature; co-expression in inflamed tissues	Possible acid-related modulation; no direct demonstration in OA (Speculative role in acid-mechanical sensing)	Limited	[30,31]

**Table 2:** Therapeutic Approaches for Targeting GPR68 in OA.

Therapeutic Strategy	Rationale/Mechanistic Basis	Key Evidence (Non-Joint Systems)	OA Relevance/Translational Opportunity
Positive Allosteric Modulators (PAMs)/Agonists (e.g., Ogerin)	Enhance proton-dependent GPR68 activity; promote Gs-cAMP-CREB signaling; reverse myofibroblast differentiation	Ogerin identified as first GPR68 PAM [57]; suppresses fibrosis and promotes stromal reprogramming [58]	May support adaptive stress responses in chondrocytes; potential anti-fibrotic effects in synovium; relevance depends on compartment-specific GPR68 function and remains untested in OA models
GPR68 Antagonists/Inhibitors (e.g., Ogremorphin)	Block GPR68-mediated Ca^2+^, MAPK, and survival pathways; inhibit acidic microenvironment–driven signaling	Ogremorphin inhibits GPR68 to block migration/metastasis [59,60]; promotes ferroptosis and radiosensitization in cancer [59,60,61]; reduces mucin expression in airway epithelium [59,60]	Could suppress inflammatory, chemotactic, or pro-survival GPR68 signaling in synovium or macrophages; requires local delivery due to vascular effects [30]; directionality and net benefit in OA remain unresolved
Local Intra-Articular Delivery	Achieves high joint concentration, minimizes systemic effects	Responsive hydrogels and nanoparticles for OA therapy [63,64,65]; MOF nanoparticles selectively target inflamed acidic tissues [66]	Optimal for GPR68 due to joint-specific acidity; allows compartment-targeted modulation (cartilage vs. synovium); conceptual strategy not yet tested with GPR68 modulators in OA
pH-Responsive Delivery Systems	Release drug preferentially in acidic microdomains (pH 6.5–6.9), matching GPR68 activation window	HA-modified MOF nanoparticles deliver therapeutics to low-pH cartilage and synovium [66]; pH-targeted liposomes release cargo in acidic OA joints [67]	Enables selective activation or inhibition of GPR68 where pH is pathologically reduced; supports hypothesis-driven targeting rather than validated OA therapy
Pharmacodynamic (PD) Biomarkers	Needed to measure GPR68 pathway engagement in vivo	pH mapping with acidoCEST-UTE MRI [13,15]; inflammatory markers (IL-6, CCL2, PGE_2_) regulated by GPR68 pathways [38,39,43]	Allows patient stratification (“acid-high OA”), dose optimization, and early trial readouts; proposed biomarkers require validation for GPR68-specific effects in OA
GPR68 Endotype-Guided Therapy	Acidification and GPR68 expression vary across patients	Evidence of OA endotypes based on pH [15], cytokine signatures [54], and fibroblast activation pathways [47,52]	Supports precision medicine approaches targeting GPR68 in acid-high, inflammation-prone OA joint environments; currently conceptual and hypothesis-generating

## Data Availability

No new data were created or analyzed in this study. Data sharing is not applicable to this article.
